# Error in Nonauthor Collaborators List

**DOI:** 10.1001/jamanetworkopen.2022.49107

**Published:** 2022-12-14

In the Original Investigation titled “Adjunctive Transdermal Cannabidiol for Adults With Focal Epilepsy,”^1^ published July 8, 2022, there was an error in the nonauthor collaborators list. One of the nonauthor collaborators was missing. This article has been corrected.^1^
